# Targeting the Oncoprotein Smoothened by Small Molecules: Focus on Novel Acylguanidine Derivatives as Potent Smoothened Inhibitors

**DOI:** 10.3390/cells7120272

**Published:** 2018-12-14

**Authors:** Silvia Pietrobono, Barbara Stecca

**Affiliations:** Tumor Cell Biology Unit–Core Research Laboratory, Institute for Cancer Research, Prevention and Clinical Network (ISPRO), 50139 Florence, Italy; silvia.pietrobono@ittumori.it

**Keywords:** hedgehog, smoothened, missense mutations, small molecule inhibitors, GLI, cancer, targeted therapy, drug-resistance, acylguanidine derivative

## Abstract

Hedgehog-GLI (HH) signaling was originally identified as a critical morphogenetic pathway in embryonic development. Since its discovery, a multitude of studies have reported that HH signaling also plays key roles in a variety of cancer types and in maintaining tumor-initiating cells. Smoothened (SMO) is the main transducer of HH signaling, and in the last few years, it has emerged as a promising therapeutic target for anticancer therapy. Although vismodegib and sonidegib have demonstrated effectiveness for the treatment of basal cell carcinoma (BCC), their clinical use has been hampered by severe side effects, low selectivity against cancer stem cells, and the onset of mutation-driven drug resistance. Moreover, SMO antagonists are not effective in cancers where HH activation is due to mutations of pathway components downstream of SMO, or in the case of noncanonical, SMO-independent activation of the GLI transcription factors, the final mediators of HH signaling. Here, we review the current and rapidly expanding field of SMO small-molecule inhibitors in experimental and clinical settings, focusing on a class of acylguanidine derivatives. We also discuss various aspects of SMO, including mechanisms of resistance to SMO antagonists.

## 1. Introduction

As one of several morphogenetic signaling pathways, Hedgehog-GLI (HH) signaling is essential for developmental processes and organ homeostasis, but its aberrant activation drives tumorigenesis [1]. Development of therapeutics for HH signaling has primarily focused on targeting Smoothened (SMO) and GLI. Natural and synthetic antagonists have been developed for SMO, and many have undergone clinical trials with varying degrees of success. SMO inhibition was first characterized through binding studies of cyclopamine [2,3], a natural steroidal alkaloid derived from *Veratrum californicum*. Derivatives of cyclopamine have been developed with the aim of increasing specificity and pharmacological potency while limiting side effects. A setback in targeting SMO has been the observation of spontaneous mutations that can develop as a response to some SMO inhibitors (SMOi). In the last few years, the transcription factor GLI1, the best-characterized downstream mediator of HH signaling and a target itself of the pathway, has also emerged as a therapeutic target. However, the list of specific GLI antagonists is not as extensive as for SMO and none of them is in clinical trials. Here, we review SMO small-molecule inhibitors used in clinical trials and preclinical studies. In particular, we focus on a class of acylguanidine derivatives as novel and potent SMO inhibitors, attempting to provide a basis for future studies and development. Various aspects of SMO are discussed, including its structure, the emergence of resistance-associated SMO mutations, and mechanisms of acquired resistance to SMO antagonists in cancer.

## 2. Hedgehog-GLI Signaling Pathway at a Glance

**Figure 1:**
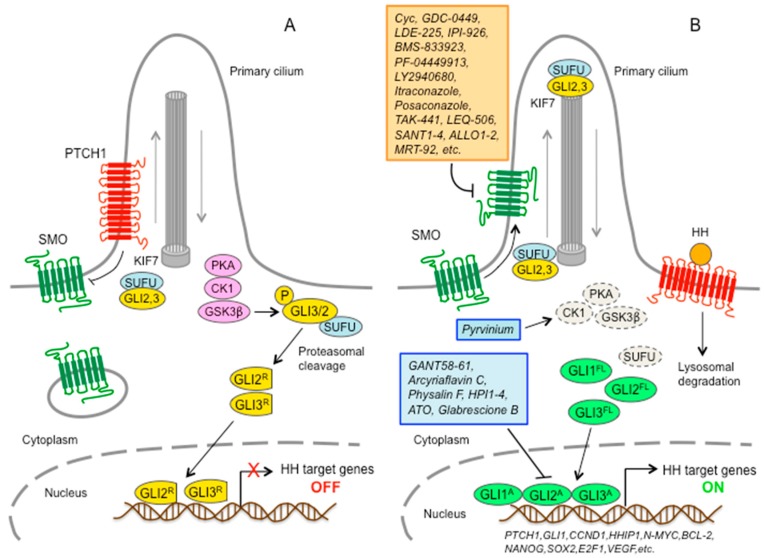
Overview of the Hedgehog-GLI pathway with SMO and GLI antagonists. In absence of HH ligands (**A**), PTCH1 inhibits SMO by preventing its entry into the primary cilium (PC). GLI2 and GLI3 proteins are sequestered in the cytoplasm by SUFU and phosphorylated by PKA, GSK3β, and CK1, which create binding sites for the E3 ubiquitin ligase β-TrCP (β-Transducin Repeat-Containing Protein). GLI3 and, to a lesser extent, GLI2 undergo partial proteasome degradation, leading to the formation of repressor forms (GLI3^R^/2^R^) that translocate into the nucleus where they inhibit the transcription of HH target genes. Upon HH ligand binding (**B**), PTCH1 is displaced from the PC, allowing accumulation and activation of SMO. Active SMO relieves SUFU-mediated suppression of GLI2 and GLI3 within the PC. GLI2 and GLI3 maintain their full-length status and bypass phosphorylation. Activator forms of GLI (GLI1^A^/2^A^/3^A^) translocate into the nucleus, where they induce the transcription of HH pathway target genes. Movement of GLI2 and GLI3 within the PC occurs in conjunction with KIF7, a member of the kinesin family of anterograde motor proteins. SMO (orange box) and GLI (light blue box) antagonists are indicated in (**B**). GLI inhibitor Pyrvinium enhances CK1α-dependent degradation of GLI^A^. CK1, casein kinase 1; GSK3β, glycogen synthase kinase 3β; PKA, protein kinase A; PTCH1, Patched 1; SMO, Smoothened; SUFU, Suppressor of Fused; HH, Hedgehog; KIF7, kinesin family member 7; ATO, arsenic trioxide.

In mammals, the core components of the HH pathway include three secreted HH ligands (Sonic (SHH), Desert (DHH), and Indian (IHH) hedgehog), the 12-pass transmembrane receptor Patched 1 (PTCH1), the 7-pass transmembrane G-protein-coupled receptor (GPCR) SMO, and three zinc-finger transcription factors (GLI1, GLI2, and GLI3) [1]. Many of these components are localized in the primary cilium, a solitary organelle that protrudes from the cell surface of most mammalian cells and functions as the core transduction machine of HH signaling [4] (Figure 1).

A simplified model posits that in absence of HH ligands, PTCH1 localizes to the primary cilium, where it inhibits ciliary accumulation of SMO [5]. As a consequence, GLI2 and GLI3 are sequestered in the cytoplasm by Suppressor of Fused (SUFU); phosphorylated by protein kinase A (PKA), casein kinase 1 (CK1), and glycogen synthase kinase 3β (GSK3β); and then processed by the proteasome into C-terminally truncated repressor forms (GLI2^R^ and GLI3^R^) [6,7,8] that act as repressors of transcription. Upon binding of the HH ligand, PTCH1 exits the primary cilium, thus releasing the inhibition on SMO and allowing the translocation of SMO into the primary cilium. Active SMO initiates an intracellular signaling cascade that promotes activation of GLI2 and GLI3. Above all, dissociation of GLI2 and GLI3 from SUFU results in fully activated GLI2 and GLI3 (GLI2^A^ and GLI3^A^), which translocate into the nucleus and turn on transcription of HH pathway target genes, including GLI1.

The three GLI transcription factors are members of the Kruppel family and they all share five conserved C2H2 zinc-finger DNA binding domains and a histidine/cysteine linker sequence between zinc fingers. GLI factors recognize the consensus sequence 5′-GACCACCCA-3′ on the promoter of target genes [9], although they can bind to variant GLI binding sites with lower affinity but still leading to strong transcriptional activation [10]. The three GLI also share a C-terminal activation domain; however, only GLI2 and GLI3 contain N-terminal repressor domains. Therefore, GLI2 and GLI3 act as activators of transcription in their full-length forms or as distinct repressor forms when truncated by processing, whereas GLI1 encodes an activator that amplifies the response of the HH pathway. Direct targets of GLI include cell fate determinants of tissue patterning, factors involved in regulation of cell proliferation and differentiation, survival, angiogenesis, self-renewal, epithelial-mesenchymal transition, and invasiveness. Among the targets, there are also GLI1 itself, which further amplifies the initial HH signaling, and the HH pathway negative regulators PTCH1 and hedgehog-interacting protein (HHIP1), which restrain HH signaling (Figure 1).

### Mechanisms of Hedgehog Pathway Activation in Cancer

While controlled HH signaling activity is a prerogative of tissue repair and homeostasis, abnormal activation of the pathway is implicated in a variety of cancers, including those of the skin, brain, lungs, prostate, breast, gastrointestinal tract, and hematologic malignancies. Several mechanisms for aberrant activation of HH signaling have been described.

**Figure 2:**
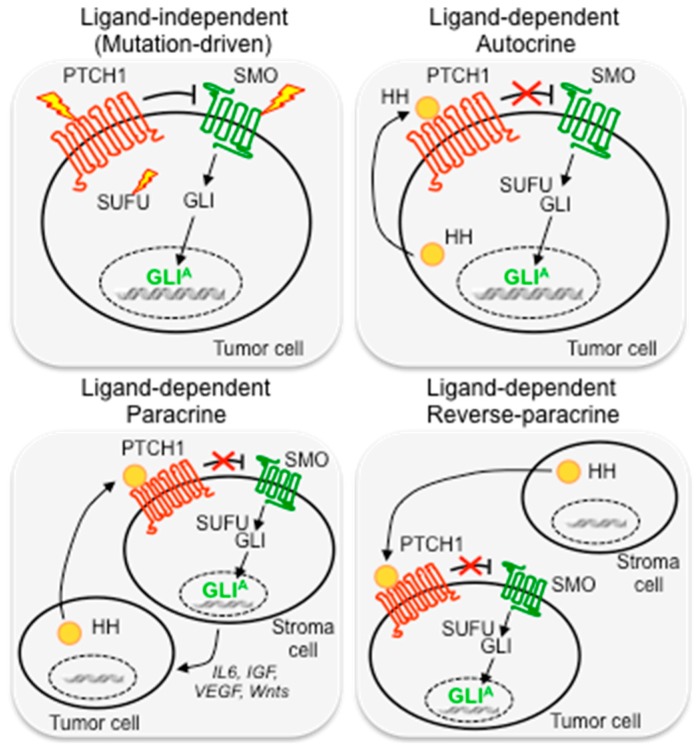
Mechanisms of Hedgehog pathway activation in cancer. Ligand-independent activation is due to inactivating mutations in the negative regulators PTCH1 or SUFU, activating mutations in SMO, or amplification of GLI activators. Ligand-dependent activation occurs through autocrine, paracrine, or inverse paracrine mechanisms (see text for details). In the autocrine mechanism, tumor cells secrete and respond to HH ligands; in the paracrine pattern tumor cells produce HH ligands, which activate HH pathway in stroma cells; in the reverse-paracrine mechanism stroma cells produce HH ligands, which activate HH pathway in tumor cells. PTCH1, Patched 1; SMO, Smoothened; SUFU, Suppressor of Fused; HH, hedgehog ligand; GLI^A^, GLI activators; IL6, Interleukin-6; IGF, Insulin Growth Factor; VEGF, Vascular Endothelial Growth Factor; Wnt, Wingless/Integrated.

Ligand-independent activation refers to mutations or amplifications of key components of the HH pathway, which induce constitutive HH pathway activation, such as loss-of-function mutations in the negative regulators PTCH1 [11,12] or SUFU [13], activating mutations in SMO [14], or GLI1 and GLI2 gene amplifications [15,16]. This type of activation occurs in a distinct set of solid tumors, such as basal cell carcinoma (BCC), medulloblastoma (MB), as well as rhabdomyosarcoma. GLI3 missense mutations of unknown pathogenic effect and copy gain have also been described in pancreatic cancers and in primary cutaneous melanomas [17,18] (Figure 2).

The ligand-dependent mechanism is another important mode of aberrant activation of HH signaling in cancer, characterized by the presence of HH ligands that activate the pathway. According to the secreted pattern of HH ligands, it can be autocrine, paracrine, and reverse paracrine. In the autocrine pattern, tumor cells secrete and respond to HH ligands. This type of activation has been described in several types of cancer, including lung, pancreas, gastrointestinal tract, prostate and colon cancer, glioma and melanoma, as well as in cancer stem cells [19,20,21,22,23,24,25,26,27,28]. In the paracrine pattern, a mode of action that resembles the physiological HH signaling during development, HH ligands secreted by cancer cells activate HH signaling in the surrounding stroma. Evidence supporting this mechanism has been revealed from studies in human tumor xenograft models of pancreatic and colorectal cancers [29]. Similarly, reverse paracrine HH pathway activation, in which HH ligands are secreted by the tumor microenvironment and activate the pathway in tumor cells, has been described in an experimental model of glioma [30] and in hematological malignancies, such as B-cell lymphoma and mantle cell lymphoma [31,32] (Figure 2).

In addition to canonical signaling, an increasing number of reports indicate that distinct tumorigenic inputs and signaling pathways can influence the activity of the GLI transcription factors independently of upstream HH ligands or PTCH1/SMO. For instance, RAS-RAF-MEK-ERK and PI3K/AKT signaling have been shown to stimulate GLI1 activity in normal murine fibroblasts and melanoma cells [28,33]. Similarly, in a mouse model of mutant K-Ras-induced pancreatic tumorigenesis, SMO deficiency does not alter tumor formation. Furthermore, mutant K-Ras cells induce SMO-independent activation of GLI1, which is required for the survival of K-Ras-transformed pancreatic cancer cells [34]. In esophageal adenocarcinoma, tumor necrosis factor-α (TNF-α)-induced activation of the mammalian target of rapamycin (mTOR)-S6 kinase 1 (S6K1) pathway promotes GLI activity in an SMO-independent manner through phosphorylation of GLI1 [35]. Furthermore, the oncogenic WIP1 phosphatase enhances GLI1 activity and stability in melanoma cells [36]. In addition, GLI proteins can be negatively regulated by tumor suppressors. For instance, in glioblastoma cells, p53 inhibits GLI1 expression, protein activity, and nuclear localization [37]. In turn, GLI1 inhibits p53 by activating MDM2 [37,38]. Furthermore, inactivation of SNF5, a tumor suppressor of the SWI/SNF chromatin remodeling complex, leads to constitutive noncanonical activation of GLI1 in malignant rhabdoid tumors [39]. It is becoming increasingly clear that noncanonical mechanisms of HH pathway activation play important roles in both tumor initiation and progression.

## 3. Smoothened: Structure of the Receptor and Mutations in Cancer

**Figure 3:**
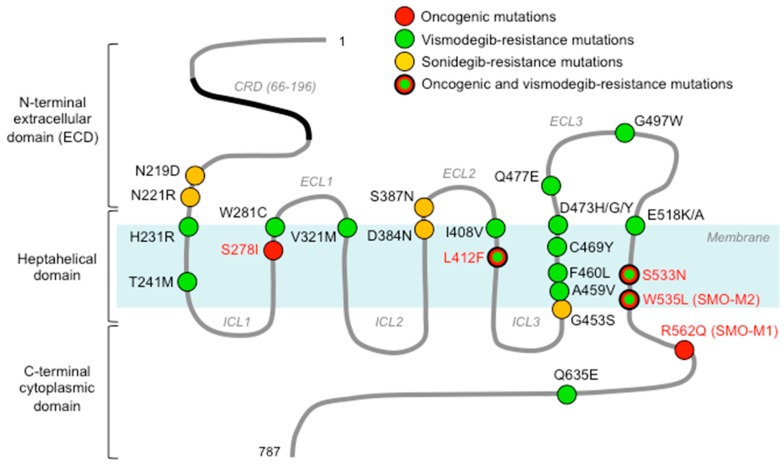
Schematic structure of the human SMO protein, showing the location of oncogenic mutations (red), vismodegib-resistance mutations (green), sonidegib-resistance mutations (orange) and oncogenic mutations associated with vismodegib resistance (red, green and black). Numbers represent amino acids. Human SMO contains 787 amino acids organized in three main domains: the N-terminal extracellular domain (ECD) (residues 1–220), containing a cysteine-rich domain (CRD); the heptahelical membrane spanning (7-TM) domain (TMD) (residues 221–558); the C-terminal cytoplasmic domain (residues 559–787). The TMD consists of seven transmembrane domains connected by three extracellular loops (ECL1–3) outside of the plasma membrane and three intracellular loops (ICL1–3). See Table 1 for details about mutations.

SMO belongs to the superfamily of GPCR, the largest class of cell-surface receptors in vertebrates, most closely related to the Frizzled family (class F) of Wnt receptors [40]. In humans, SMO comprises 787 amino acids organized in three main domains: (1) an N-terminal extracellular domain (ECD) (residues 1–220) constituted by a cysteine-rich domain (CRD), a linker domain, and a hinge domain [41]; (2) an heptahelical membrane spanning (7-TM) domain (TMD) typical of all GPCRs (residues 221–558) [42]; and (3) a less characterized C-terminal cytoplasmic domain (residues 559–787) that has been related to HH pathway inhibition in *Drosophila* [43] (Figure 3).

Several features of SMO resemble those of other GPCRs, although SMO uses PTCH as the receptor for secreted HH ligands instead of directly interacting with it. First, post-transcriptional modifications, such as phosphorylation, control the switch between on/off signaling states of SMO. For instance, in *Drosophila* Smo (dSmo), activation requires phosphorylation of its cytoplasmic tail by PKA, CK1, and GSK3β [44], whereas the G protein-coupled receptor kinase 2 (Gprk2) has been shown to activate vertebrate Smo (vSmo) by promoting its internalization [45]. Of note, four clusters of phosphorylation sites for Gprk2 in the membrane-proximal C-terminus of SMO have been shown to enhance its dimerization and activity [46]. Phosphatidylinositol 4-phosphate (PI4P) has been also shown to directly interact with SMO through an arginine motif in the SMO C-terminal tail, promoting its phosphorylation, activation, and ciliary localization [47]. Second, SMO can form homodimers through its cytoplasmic tail and undergo a large conformation change in response to its activation [48,49]. Third, SMO can signal through heterotrimeric G-proteins, as missense mutations in residues necessary for G-protein coupling generate an SMO loss-of-function phenotype (i.e., R474C mutation in the third intracellular loop of TMD in dSmo [50] or W535L mutation in the TMD of human SMO [14]). Early work in *Xenopus* melanophores supported the requirement of G proteins in Smo signaling, showing that ectopic expression of human SMO induces a phenotype of persistent pigment aggregation by signaling through the α-subunit of the G protein, G_i_ [51]. Additionally, constitutive activation of the G_12_ family of heterotrimeric G proteins has been shown to induce transcriptional activation of GLI1 [52]. On the contrary, previous work by Riobò et al. showed that mammalian SMO activates all members of the G_i_ family but not the G_12_ family, driving GLI activation in fibroblasts [53]. A recent study showed that the C-terminus of SMO recruits the ubiquitin ligase complex Cullin4–DNA damage binding protein 1 (Cul4-DDB1) through the β subunit of G protein (Gβ), which promotes ubiquitination of both SMO and Gprk2 and, hence, internalization and degradation of SMO [54].

### 3.1. Structure of Smoothened

Recent X-ray diffraction and nuclear magnetic resonance spectroscopy studies allowed the characterization of both TMD and CRD of SMO. The TMD consists of a 7-TM connected by three extracellular loops (ECL1–3) outside of the plasma membrane and three intracellular loops (ICL1–3) [42]. The ECL integrity is indispensable for maintaining the inactive state of SMO, as mutations in cysteine residues increase its activity [55]. A short intracellular helix 8, which is located between the transmembrane (TM) helix 7 (TM7) and the C-terminal domain and runs parallel to the membrane, together with a helical turn in the short ICL1, favors an overall spatial conformation of the TMD typical of all class A GPCRs [56]. The CRD is stacked above the 7-TM, with a small wedge-like linker domain that connects the CRD to the TM1 and to the extended ECL3 located between TM6 and TM7 helices. Hydrophobic interactions between the CRD and both the ECL3 and the linker domain appear crucial for the positioning of CRD on the plasma membrane. Nine disulphide bonds stabilize the global architecture of this complex and appear conserved within class F GPCRs [42].

Unlike most class A GPCRs, Smoothened is characterized by the absence of most conserved prolines in the NPXXY motif. It shows an enrichment of glycine residues in the abovementioned helices important for their bending and 7-TM packaging, and a cluster of tryptophan residues conserved within the class F GPCRs (W331 and W339 in helix 3, W365 in helix 4, and W535 in helix 7) [42]. In the absence of activating stimuli, the inactive conformation of SMO is locked by interactions that ensure the TM5/TM6 closed state, similar to class F GPCRs. In particular, the presence of the cation-π lock between the aromatic electron density of the tryptophan residue in TM7 (W535 in human SMO and W539 in mouse SMO) and the positively charged guanidine group of an arginine residue in TM6 prevents the outward opening of TM6 necessary for activation. Ligand binding breaks the cation-π lock and induces a severe reorientation of the CRD, which moves closer to the membrane. This rotation is then transmitted to the TMD, which undergoes to the outward movement of TM6 and TM5 helices, with the consequent opening of a cavity on the cytoplasmic side of SMO that allows the switch toward the active conformation (TM5/TM6 open state) [57]. Several natural and synthetic small molecules modulate the function of SMO by binding to its TMD [42,58,59] (see below).

### 3.2. Smoothened Binding Sites

SMO appears regulated by two small-molecule binding sites, one in the TMD and one in the CRD. Both synthetic SMO agonists (SAG1.5 and purmorphamine) and antagonists (i.e., cyclopamine, LY2940680, ANTA XV, SANT-1, and vismodegib) were found to bind to a thin pocket constituted by ECL protrusions and the extracellular stretch of the TMD [42,58,60,61,62,63,64]. This drug-binding pocket (DBP) is exposed in the extracellular compartment and allows drugs to access the cavity from the extracellular space, with their axes perpendicular to the membrane. However, the depth of entry differs between these small molecules, as SAG1.5, cyclopamine, LY2940680, and ANTA XV interact mainly with the ECLs, lining the top of the ligand-binding cavity, whereas SANT1 contacts ECL2 within the TMD and binds deeply in the 7-TM helical bundle [42,58,59,64].

Several mutational studies of SMO allowed the identification of residues involved in the interaction between SMO and small molecule regulators. First, the SMO agonist SAG1.5 has been found to interact with three key residues important for SMO remodeling and activation: R400 (helix 5), D473 (helix 6), and E518 (helix 7) [58]. Importantly, D473 and E518 residues were found to interact with vismodegib [65] and ANTA XV [58], as their mutations (D473H/A and E518A) are associated with drug resistance [58,65] (see below), underlying the importance of chemical modifications on these compounds to overcome drug-resistant D473 mutations [66,67]. Conversely, LY2940680 shows only weak interactions with D473, despite sharing a similar scaffold with ANTA XV [42], and D473 mutations do not interfere with its binding within the DBP of SMO [68]. Indeed, the function of LY2940680 appears to require the interaction with Q477, W480, E481, and F484 residues [58]. Unlike other SMO antagonists, SANT-1 has been shown to bind deeper within the binding pocket by interacting with residues 329, 408, and 466 of SMO, as revealed by a substantial reduction of SANT-1 binding after the introduction of mutations within these residues (V329F, I408F, and T466Q) [58].

A second binding site of SMO appears to bind cholesterol, hydrooxysterols such as 20(S)-hydroxycholesterol (20(S)-OHC) in vSmo [64,69,70,71], and glucocorticoids in both vSmo and dSmo [72], which have been shown to activate the HH pathway even in the absence of HH ligands and to promote SMO relocalization to the primary cilium in mammalian cultures [64,69,70], with the exception of vitamin D3 [73]. These molecules have been suggested to function as endogenous SMO ligands that bind a domain different from the 7-TM orthosteric site shown to interact with the abovementioned SMO agonists and antagonists. This domain is conserved in all class A GPCRs [74,75,76]. Recent reports indicate that sterols occupy a hydrophobic groove delimited by two α-helices in the ECD. This binding reduces the flexibility of CRD structure and stabilizes SMO in an inactive conformation that is able to respond to HH signals [64]. SMO variants deleted in their ECD (SmoΔCRD) show a higher basal activity than full-length SMO, denoting a repressive role of unbounded CRD on the TMD [64,77].

### 3.3. Oncogenic Smoothened Mutations

Several studies demonstrated that a number of gain-of-function mutations of SMO are implicated in the pathogenesis of cancers such as BCC and MB. These missense mutations fall into residues critical for enabling conformational changes between active and inactive states, leading to constitutive activation of the receptor (Table 1).

Two mutations were previously reported to lead to constitutive activation of SMO in sporadic BCC: R562Q (SMO-M1) and W535L (SMO-M2) [14,83]. W535 mutation has been identified also in meningiomas [82] and ameloblastomas [81]. The W535 residue is located at the intracellular end of helix 7, parallel to the membrane layer and adjacent to helix 8, and represents a critical player during SMO activation. Other oncogenic mutations of SMO that are associated with constitutive HH pathway activation include: L412F, which has been reported in desmoplastic MB [88], meningiomas [82], and ameloblastomas, where it has been associated with insensitivity to vismodegib [81]; S533N in primitive neuroectodermal tumors (PNET) [83]; and S278I in both BCC and MB [78,79]. Interestingly, W535L and S533N are located outside of the DBP of SMO and are believed to alter the conformation of SMO in order to prevent the access of small-molecule antagonists to the SMO DBP [87].

## 4. Smoothened Inhibitors

SMO is the primary target for the development of HH pathway inhibitors. Inhibition of SMO hinders downstream activation of GLI transcription factors, leading to repression of target genes associated with tumor growth and progression. Table 2 summarizes SMO antagonists, with the proposed mechanism of action and preclinical/clinical status.

The first identified SMO antagonist was cyclopamine, a steroidal alkaloid derived from *V. californicum* with teratogenic properties [89], that showed great potential to bind SMO and inhibit the HH pathway [2,90]. Binding studies using a fluorescent cyclopamine derivative suggested that cyclopamine binds to the TMD of SMO, preventing the conformational shift necessary to activate SMO [3]. Cyclopamine has been widely used as an HH inhibitor with promising outcomes in a variety of mouse xenograft models of human cancers, including MB, glioma, melanoma, colon, pancreatic, and prostate cancers [20,23,25,26,28,91]. However, poor oral solubility and severe side effects in mice prevented further clinical development of cyclopamine. Efforts to improve the specificity, potency, and pharmacologic profile of cyclopamine have led to the synthesis of derivatives such as KAAD-cyclopamine [3] and IPI-269609 [92]. In recent years, many SMO inhibitors have been generated and tested in preclinical models and clinical trials and showed variable degrees of efficacy as anticancer agents.

### 4.1. SMO Inhibitors in Clinical Trials

#### 4.1.1. Vismodegib (GDC-0449)

Vismodegib is a second-generation cyclopamine derivative that binds to the 7-TM pocket of SMO, preventing downstream GLI activation. Vismodegib was the first HH pathway inhibitor to be approved by U.S. Food and Drug Administration (FDA). Vismodegib is currently being used for treatment of advanced and metastatic BCC [93]. Although a number of preclinical and clinical studies conducted in BCC and MB patients have documented a significant initial efficacy of the treatment [93,94,95,96], use of vismodegib has been invariably associated with the appearance of unique SMO mutations (Table 1; Figure 3) and occurrence of compensatory mechanisms that confer resistance to this drug (see below) [65,97]. Currently, vismodegib is being studied as monotherapy and in combination with other chemotherapeutics in a long list of clinical trials in a wide array of cancers, including BCC, MB, small-cell lung cancer, metastatic pancreatic and prostate cancer, meningioma, recurrent glioblastoma, and acute myeloid leukemia.

#### 4.1.2. Sonidegib (Erismodegib, LDE-225, NVP-LDE225)

Sonidegib was approved by the FDA in July 2015 to treat adult patients with locally advanced or metastatic BCC, becoming the second HH pathway inhibitor receiving FDA approval. Sonidegib was shown to reduce tumor growth in a mouse model of MB and in xenografts of human prostate cancer stem cells and of human melanoma cells [84,138,139]. Acquired resistance to sonidegib in a mouse model of MB was associated with mutations in mouse SMO, including N223D, L225R, D388N, S391N, and G457S [84], residues conserved in human SMO (Table 1; Figure 3). Several phase I and II trials for sonidegib in monotherapy and in combination are currently underway in both solid and hematological malignancies [140,141,142,143].

#### 4.1.3. Saridegib (IPI-926)

Modification of the cyclopamine A-ring system led to the discovery of the D-homocyclopamine analogue saridegib (IPI-926), which showed improved pharmaceutical properties and potency and a more favorable pharmacokinetic profile compared to cyclopamine and IPI-269609 [100]. Saridegib was shown to reduce growth of medulloblastoma allografts [100] and chondrosarcoma xenografts [144] and to prolong survival in an aggressive *Ptch1*-null medulloblastoma model [145]. It also enhanced delivery and efficacy of chemotherapy in a mouse model of pancreatic cancer [146]. Saridegib is a substrate of the P-glycoprotein (Pgp) transporter, which mediates the drug efflux from tumor cells. Since saridegib increases the expression and activity of Pgp, drug resistance may occur after an extended period of treatment [145]. Interestingly, saridegib is active in cells with the D473H point mutation that renders them resistant to vismodegib [145]. Although saridegib showed desirable preclinical absorption, distribution, metabolism, and excretion properties [147], and a phase I study in adult patients with solid tumors demonstrated a good pharmacokinetic profile [148], it has been discontinued for lack of response [149,150].

#### 4.1.4. BMS-833923 (XL139)

BMS-833923 is an orally bioavailable and small-molecule antagonist of SMO that has been found to reduce *GLI1* and *PTCH1* mRNA expression in vitro and to decrease cell proliferation in human cholangiocarcinoma cells and esophageal carcinoma cell lines [101,151,152]. Phase I clinical trials were completed in basal cell nevus syndrome, multiple myeloma, gastrointestinal cancer, and small-cell lung cancer, but the results are not available.

#### 4.1.5. PF-04449913 (Glasdegib)

PF-04449913 is a potent and selective HH pathway inhibitor that binds SMO and blocks signal transduction [102]. Treatment with PF-04449913 decreased the initiation potential of acute myeloid leukemia (AML) cells in a serial transplantation mouse model, reduced tumor burden, and sensitized AML cells to cytosine arabinoside [153]. It is currently in clinical trials for treatment of hematological malignancies [154,155,156].

#### 4.1.6. LY2940680 (Taladegib)

LY2940680 is an orally bioavailable potent small molecule that inhibits HH signaling in Daoy medulloblastoma cells and medulloblastoma growth in *Ptch*^+/−^; *p53*^−/−^ mice. Importantly, LY2940680 was shown to inhibit the activity of the vismodegib-resistant SMO-D473H mutant [68]. LY2940680 binds to the extracellular end of the 7-TM bundle of SMO [42]. Currently, LY2940680 is being tested in phase I and II trials for advanced solid tumors, including treated-naive and previously treated BCC [157].

#### 4.1.7. TAK-441

TAK-441 was first described as a highly potent and orally bioavailable SMO inhibitor [108]. Reports of its inhibition have been found against MB, pancreatic cancer, and prostate cancer. In a prostate cancer xenograft mouse model, TAK-441 seems to delay castration-resistant progression by suppressing paracrine HH signaling [158]. It is also effective in inhibiting the vismodegib-resistant SMO-D473H mutant [159]. A phase I clinical trial in patients with advanced nonhematological malignancies was completed, and TAK-441 has been discontinued for lack of response [160]. 

#### 4.1.8. LEQ-506

LEQ-506 shows efficacy in preventing proliferation of a cell line carrying an SMO-D473H mutation that confers resistance to vismodegib (see below). LEQ-506 is effective in reducing MB growth in animals xenografted with primary tumors from *Ptch1*^+/−^ mice [67]. Phase I clinical studies in advanced solid tumors were completed, but results were not disclosed.

#### 4.1.9. Vitamin D3

Vitamin D3 has been shown to bind SMO with high affinity in a cyclopamine-sensitive manner. Treatment of zebrafish embryos with vitamin D3 mimics the *smo*^−/−^ phenotype, confirming its inhibitory action in vivo [73]. Vitamin D3 showed significant antiproliferative activity and ability to reduce *GLI1* expression in a HH-dependent mouse model of BCC [161], offering promises as an effective anti-BCC agent. However, results of a phase II clinical trial for topical administration of calcitrol (a vitamin D3 analogue) in combination with the nonsteroidal anti-inflammatory drug diclofenac showed a lack of clinical effectiveness for calcitrol in superficial BCC [162]. Vitamin D3 is currently in two phase I clinical trials as neoadjuvant for treatment of BCC.

### 4.2. SMO Inhibitors in Preclinical Studies

A number of additional SMO antagonists have been used in preclinical studies (Table 2). They include Cur-61414 [106,163], Sant1-4 [60], the bis-amide compound 5 [66], desmethylveramiline [164], and PF-5274857 [107]. Among them, compound 5 has been shown to inhibit tumor growth mediated by vismodegib-resistant SMO (D477G) in a murine allograft model of MB [66]. PF-5274857 was described as a potent SMO antagonist with the ability to penetrate the blood-brain barrier. Indeed, it abrogated tumor growth in a *Ptch1*^+/−^; *p53*^−/−^ MB mouse model [107]. Although preclinical studies suggested that PF-5274857 might be an ideal candidate for treatment of brain malignancies, no clinical trials to verify its effectiveness are currently underway.

An attractive alternative to prevent the occurrence of drug-resistant mutations is to use SMO inhibitors that bind to sites distinct from that of vismodegib. For instance, ALLO-1 has been reported to bind SMO at the extracellular CRD and to inhibit both WT and drug-resistant SMO mutants [108]. Similarly, the antifungal agent itraconazole, a potent inhibitor of the HH pathway, prevents ciliary translocation of SMO [103]. Systemic administration of itraconazole inhibits growth of HH-dependent MB and BCC in mice and it is also active against drug-resistant mutant SMO-D473H and GLI2 overexpression [165]. Recently, posaconazole, a second-generation triazole antifungal with minimal drug–drug interaction and a favorable side-effect profile, has shown robust inhibitory activity against drug-resistant SMO mutants and against growth of HH-dependent BCC in vivo [104]. Both itraconazole and posaconazole are currently in clinical trials for several types of cancer.

An alternative way for inhibiting oncogenic HH signaling is through interference with SMO ciliary trafficking. For instance, the glucocorticoid budesonide inhibits SMO ciliary translocation and is active against oncogenic (SMO-M2) and resistant (SMO-D473H) SMO mutants [114]. Wang and colleagues identified two HH signaling antagonists through a direct screen for inhibitors of SMO ciliary translocation. DY-131 inhibits SMO signaling through a common binding site shared by reported SMO agonists and antagonists. SMANT, on the other hand, inhibits the oncogenic form of SMO-M2 [115]. Compounds SA1–10 were found to inhibit HH signaling, with SA1–7 and SA10 specifically inhibiting trafficking of intracellular SMO to cilia. In contrast, SA8 and SA9 recruit endogenous SMO to the cilium. Despite the different mechanisms of action, all of the SAs were reported to abrogate growth of the murine ASZ1 BCC cell line and to inhibit activation of the HH pathway by the oncogenic SMO-M2 form [113].

In addition to the above, other important SMO antagonists include SEN450 [119], A8 [118], Smoothib [116], DMB5 [109], and HH78 [117]. SEN450 is a potent benzimidazole derivative that was shown to inhibit SMO and to reduce tumor volume in a glioblastoma multiforme xenograft model [119]. A8 is a compound that competes with cyclopamine for the same binding site on SMO and binds both wild-type SMO and the SMO-D473H mutant. It inhibits cell proliferation of neural precursor cells and prevents HH-signaling-dependent hair growth in mice [118]. A combination of cell-based screening and cheminformatic target prediction identified Smoothib, which was shown to target the heptahelical bundle of SMO, preventing its ciliary localization, and to suppress the growth of *Ptch*^+/−^ medulloblastoma cells [116]. Recently, a novel vismodegib analog, DMB5, was shown to bind with an extra interaction to the TMD of SMO. In a pancreatic tumor mouse model, treatment with MDB5-containing nanoparticles showed significant inhibition of tumor growth without loss in body weight [109]. HH78 was shown to displace the fluorescent SMO antagonist BODIPY-cyclopamine using U2OS cells overexpressing human SMO and to overcome vismodegib resistance in chronic myeloid leukemia cells [117].

### 4.3. Novel Acylguanidine Derivatives as Potent SMO Antagonists

**Figure 4:**
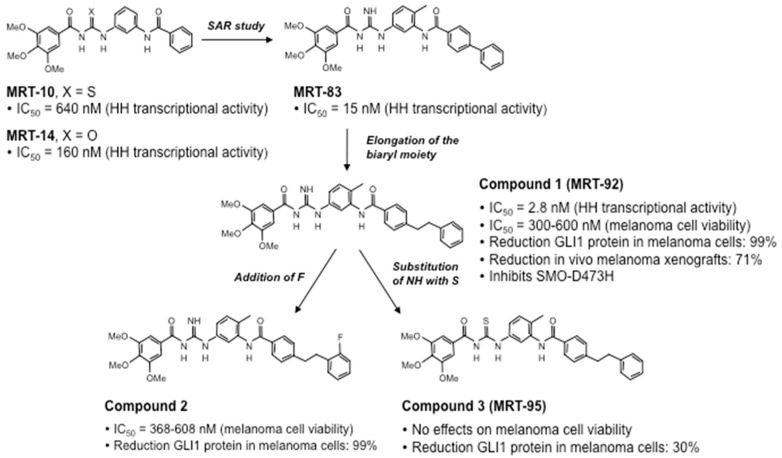
Discovery, optimization, and biological characterization of acylguanidine and acylthiourea derivatives. A virtual screening identified MRT-10 (acylthiourea) and MRT-14 (acylurea analog) as novel SMO inhibitors. Further structure-activity relationship (SAR) study yielded to the acylguanidine MRT-83, which showed nanomolar antagonist potency towards SMO. Further elongation of the biaryl moiety of MRT-83 led to acylguanidine MRT-92 (Compound 1), with a phenylethylphenyl tail. Addition of a fluorine (F) atom or substitution of the NH group with a sulphur (S) atom in MRT-92 led, respectively, to Compound 2 and Compound 3 (MRT-95). All these compounds show antagonist activity with nanomolar potency, except for Compound 3 (MRT-95), which displays much reduced activity. Inhibition of GLI1 protein expression gives an indication of the degree of HH pathway inhibition.

Recently, a novel class of SMO inhibitors based on acylthiourea, acylurea, and acylguanidine scaffolds have been developed (Figure 4). An initial virtual-screening-based discovery identified MRT-10, an SMO antagonist displaying a unique acylthiourea scaffold. MRT-10 was shown to bind to SMO at the level of the BODIPY-cyclopamine binding site. It displayed an IC_50_ value of 0.64 μM in a Shh-light 2 cell luciferase assay and the ability to inhibit SAG-induced differentiation of C3H10T1/2 cells [166]. Its acylurea analog (MRT-14) blocked BODIPY-cyclopamine binding to SMO in a dose-dependent manner and showed improved inhibitory activity against the HH pathway, with an IC_50_ value of 0.16 μM in a Shh-light 2 cell luciferase assay and increased inhibition of C3H10T1/2 cell differentiation [166].

Further structural modification yielded the acylguanidine MRT-83 (Figure 4), which showed nanomolar antagonist potency toward SMO in various HH assays, including BODIPY-cyclopamine binding to SMO and HH-mediated proliferation of cerebellum granule cell precursors (GCPs) [167]. MRT-83 did not display significant agonist or antagonist activity against Wnt signaling in HEK-293 cells transfected with a Tcf/Lef-dependent luciferase reporter, confirming the specificity toward SMO but not against the homologous Frizzled GPCRs [110]. Mechanistically, MRT-83 abrogated SAG-induced trafficking of endogenous mouse and human SMO to the primary cilium of C3H10T1/2 mouse fibroblasts and NT2 testicular carcinoma cells, respectively. Furthermore, injection of MRT-83 into the lateral ventricle of adult mice was shown to block HH-mediated PTCH transcription, demonstrating efficient inhibition of HH signaling in vivo [110].

Further elongation of the biaryl moiety of MRT-83 led to the discovery of the acylguanidine MRT-92 with a phenylethylphenyl tail (Figure 4, Compound 1), which showed to be one of the most potent SMO antagonists known so far. It displayed sub-nanomolar antagonistic activity against SMO in various HH cell-based assays, including GLI-dependent luciferase assay in Shh-light 2 cells, alkaline phosphatase activity in C3H10T1/2 cells, and proliferative activity of cerebellar GCPs [111]. Similar to MRT-83, MRT-92 did not affect the activity of the Wnt pathway. Molecular docking and site-directed mutagenesis data showed that MRT-92 binds the entire transmembrane cavity of SMO, at both the ECLs and the 7-TM bundle, making MRT-92 an SMO antagonist with a unique mode of action [111]. MRT-92 has been shown to potentially inhibit both human SMO-WT and mutant SMO-D473H with a Ki value of 0.7 nM [111].

A recent report showed that further addition of a fluorine atom to MRT-92 led to Compound 2, which showed HH inhibitory activity comparable to that of MRT-92 [112]. On the contrary, the substitution of the NH group with a sulphur atom in MRT-92 yielded the thiourea analog MRT-95 (Figure 4, Compound 3), which displayed much less potent activity than MRT-92 and MRT-83 in vitro [111,112] (Figure 4).

Interestingly, both Compounds 1 and 2 were shown to suppress melanoma cell proliferation with nanomolar IC_50_ concentrations and to reduce the expression of endogenous GLI1 protein in a dose-dependent manner. Mechanistically, Compounds 1 (MRT-92) and 2 induce a replication stress that leads to the activation of the ATR/CHK1 DNA damage signaling cascade. In particular, both compounds have been shown to bypass the G2 checkpoint, leading to the activation of mitotic catastrophe, an oncosuppressive mechanism that induces cell death in order to avoid genomic instability and cancer progression [112]. Furthermore, MRT-92 has been reported to reduce cell proliferation and induce apoptosis and autophagy in chronic myeloid leukemia cell lines, although at high micromolar concentrations [168] and in osteosarcoma cell lines [169].

MRT-92 was also shown to inhibit tumor growth in vivo. In a melanoma xenograft mouse model, MRT-92 suppressed tumor growth at a systemic dose of 15 mg/Kg and significantly decreased *GLI1* expression in tumor lesions, demonstrating efficient inhibition of HH signaling in vivo and providing the first evidence of anticancer therapeutic efficacy [112]. Likewise, oral administration of 200 mg/Kg of MRT-92 daily for two weeks inhibited by 48% in vivo tumor growth of the colorectal cancer cell line LS180 without affecting body weight and revealed an excellent ADME (absorption, distribution, metabolism, excretion) profile [170].

## 5. GLI Inhibitors

Inhibition of GLI-mediated transcription represents an alternative strategy for the development of HH pathway inhibitors and provides a good approach to block both canonical HH signaling and noncanonical activation of GLI. In addition, these inhibitors have the potential to overcome the acquired resistance of current SMO inhibitors. Thus far, only a few GLI antagonists have been identified and, except for arsenic trioxide (ATO), which is not a specific GLI inhibitor, their use has been limited to preclinical studies (Figure 1; Table 2). A cell-based screening for inhibitors of GLI1-mediated transcription identified two structurally different compounds, the hexahydropyrimidine derivatives GANT61 and GANT58, which bear a thiophene core with four pyridine rings. Both are capable of interfering with GLI1- and GLI2-mediated transcription in a dose-dependent manner and inhibit human prostate cancer xenograft growth in a GLI-dependent manner [131]. A screening of natural products identified, among others, zerumbone, arcyriaflavin C, and physalins F and B as inhibitors of GLI-mediated transcriptional activity [132]. In addition, through a screening of 122,755 compounds, four HH inhibitors were identified in multiple cell-based assays. Each inhibitor appears to act through a unique mechanism of action: HPI-1 might target post-translational modification of the GLI protein and/or interaction of GLI with a cofactor, HPI-2 and HPI-3 likely interfere with GLI2 activation via different mechanisms, and HPI-4 seems to act by disrupting ciliogenesis [133].

ATO, an already FDA-approved therapeutic for acute promyelocytic leukemia, has been found to inhibit the GLI transcription factors [134,135]. Mechanistically, ATO directly binds to GLI1 protein, inhibits its transcriptional activity [135], and blocks HH-induced ciliary accumulation of GLI2 [134]. The in vivo efficacy of ATO was demonstrated in both studies; it inhibited the growth of *Ptch*^+/−^; *p53*^−/−^ medulloblastoma allografts and Ewing sarcoma xenografts and increased survival of constitutively activated SMO transgenic mice with MB [134,135]. Although not a specific GLI inhibitor, ATO is currently in several clinical trials for cancer treatment as a single agent or in combinatorial therapy.

Pyrvinium, an FDA-approved anti-pinworm agent, has been shown to inhibit GLI activity and enhance GLI degradation in a CK1α-dependent manner [136]. Consistent with its activity on the downstream mediators of the HH signaling, pyrvinium is able to inhibit the activity of a vismodegib-resistant SMO-D473H mutant and GLI activity resulting from loss of Sufu as well as to reduce in vivo growth of *Ptch*^+/−^ MB allografts [136].

More recently, the structural requirement of GLI1 for binding to DNA was clarified and the flavonoid derivative Glabrescione B was identified as a GLI1 inhibitor. It binds to GLI1 zinc finger and impairs GLI1/DNA interaction [137]. Moreover, Glabrescione B inhibits growth of HH-dependent BCC and MB tumor cells in vitro and in vivo, showing good inhibitory activity against cancer stem cells [137].

## 6. Mechanisms of Resistance to SMO Inhibitors

**Figure 5:**
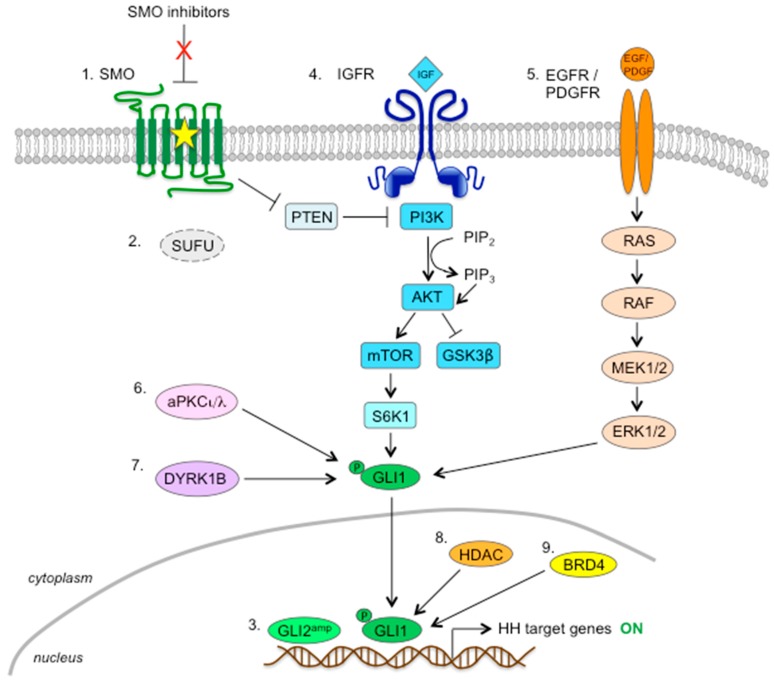
Schematic representation of the mechanisms of resistance to SMO inhibitors. These are represented by: activating mutations in SMO (1); loss of SUFU (2); amplification of *GLI2* gene (3); activation of the PI3K/AKT/mTOR pathway, which induces S6K1-dependent phosphorylation and activation of GLI1 (4); activation of the RAS/RAF/MEK/ERK pathway (5); phosphorylation- dependent activation of GLI1 by aPKCι/λ (6) or DYRK1B (7); histone deacetylases (8); BRD4 protein (9). SMO, Smoothened; SUFU, Suppressor of Fused; IGFR, Insulin growth factor receptor; PTEN, Phosphatase and tensin homolog; PI3K, Phosphatidylinositol-4,5-bisphosphate 3-kinase; mTOR, mammalian target of Rapamycin; GSK3β, Glycogen synthase kinase 3β; S6K1, Ribosomal protein S6 kinase beta-1; EGFR, Epidermal growth factor receptor; PDGFR, Platelet-derived growth factor receptor; MEK1/2, MAP (Mitogen-activated protein) Kinase/ERK (Extracellular signal-Regulated Kinase) Kinase 1; HDAC, Histone deacetylase; BRD4, Bromodomain-containing protein 4.

Although SMO inhibitors have shown promising antitumor effects against a variety of tumor types in preclinical models, several studies have reported disease progression within several months due to the acquisition of resistance. Based on these studies, three different mechanisms have been proposed to explain the acquired resistance towards these SMO inhibitors: (1) mutations in the DBP of SMO that keep it refractory to antagonist inhibition, (2) activation of the HH signaling cascade downstream of SMO, and (3) upregulation of noncanonical, compensatory signal transduction mechanisms responsible for GLI activation (Figure 5).

### 6.1. Resistance-Associated Smoothened Mutations

The first evidence of acquired resistance to SMO inhibition was described in a biopsy of a relapsed metastatic MB, in which a heterozygous G to C missense mutation in SMO at residue 1697 converted aspartate into histidine at codon 473 (D473H), not found in the pretreatment biopsy [97]. In vitro analysis of SMO-D473H showed that this substitution was not essential for SMO activity, as this mutant retained the same ability of WT SMO to transduce HH signals but made SMO insensitive to the inhibitory effect of vismodegib by abrogating its physical interaction with vismodegib [65]. Interestingly, the corresponding SMO-D473H in mice (mD477G) was found in one of the drug-resistant tumor lines originated after implantation of MB tumors arising in a *Ptch*^+/-^; *p53*^−/−^ mouse model into nude mice, followed by intermittent dosing of vismodegib [65]. Using a similar approach, 135 drug-resistant MB lines were generated from allograft mouse models after 13 days of continuous dosing of NVP-LDE-225. The analysis of these resistant tumor lines led to the identification of five missense mutations of mouse SMO different from the vismodegib-induced SMO mD477G mutation, underlining the distinct modes of action of the two SMO inhibitors. These included: mN223D (N219D), mL225R (L221R), mD388N (D384N), mS391N (S387N), and mG457S (G453S), which were responsible for resistance to NVP-LDE-225, thus suggesting that the acquisition of resistance towards SMO antagonists can result from mutations at multiple sites of SMO [84] (Table 1).

Vismodegib-resistance has also been widely documented in BCCs. The molecular mechanisms explaining drug resistance were reported for the first time in two patients, one showing loss of sensitivity to nonstop vismodegib treatment for 2 months and thus classified as a case of primary resistance, and the other starting progression 11 months after continuous treatment despite initial complete response [86]. The first case was shown to harbor a glycine-to-tryptophan substitution at codon 497 of SMO (G497W) that induced a conformational alteration able to prevent the entry of vismodegib into the DBP; the second one was characterized by an aspartate to tyrosine substitution at position 473 (D473Y) responsible for the disruption of hydrogen bonds with the nearby residues, leading to decreased affinity of vismodegib for SMO [86]. The position 473 of SMO was also found in association with a glycine substitution (D473G) in other studies on BCC [78,85], in agreement with the observation that the substitution of D473 with any other amino acid is responsible for reduced affinity of vismodegib for its target SMO [66]. Interestingly, vismodegib-resistant patients with advanced BCC carrying a D473H mutation were also found resistant to NVP-LDE-225 [87].

Genomic analyses of resistant BCCs led to the identification of several additional SMO mutations related to vismodegib resistance, which were mapped within or in the proximity of the DBP, as well as at more distant sites [78,80,85]. SMO mutations localized at DBP were almost all undetectable in the matched untreated tumors, consistently with their pivotal role in drug binding, including H231R, Q477E, Q635E [78], W281C, V321M [78,80,171], I408V, and C469Y [80]. SMO mutations localized outside of DBP were also found associated with vismodegib resistance [78,80,85]. Some of them have been previously described as oncogenic, including L412F, S533N, and W535L [14,81,172], whereas V321M and F460L have been suggested to enable SMO activation through conformational changes [78].

### 6.2. Activation of HH Pathway Downstream of SMO

Several genetic alterations in HH pathway components downstream of SMO, such as loss of SUFU, amplification of *GLI2* (Figure 5, mechanisms 1–3), and duplication of the HH signaling target gene cyclin D1 (*CCND1*), have been shown to contribute to the acquisition of resistance towards SMO inhibitors. Germline loss of the tumor suppressor SUFU has been shown to confer primary resistance to vismodegib in pediatric MB patients [173]. In another study, loss of SUFU in stable Shh-subtype MB cell lines reactivated the HH pathway downstream of SMO, causing acquired therapeutic resistance [174]. Consistently, tumor biopsies of BCC showed a 10q deletion containing *SUFU*, which was associated with the partial loss of SUFU function but was not sufficient to drive resistance to vismodegib in the absence of other co-occurring alterations, such as focal *GLI2* amplifications [80]. Amplification of chromosomal regions containing *GLI2* was also found in a model of vismodegib resistance [66], as well as in two of three sonidegib-resistant MB tumors [84], in which the increased expression of *GLI2* mRNA has been shown to mediate tumor growth independently from SMO. Importantly, silencing of *GLI2* in these tumor lines has been shown to partially restore sensitivity to the inhibitory effects of sonidegib [84]. Although not directly correlated with resistance to SMO inhibitors, increasing levels of GLI2 have also been correlated with reduced sensitivity to sonidegib in melanoma cells [175]. Additional mechanisms of acquired resistance found in MB include amplification of *CCND1* [66,176], indicating the importance of alterations in HH pathway components downstream of SMO in driving pathway activation in resistant tumors.

### 6.3. Noncanonical, Compensatory Oncogenic Signaling Pathways

The therapeutic efficacy of SMO targeting is limited due to pre-existing and acquired drug resistance. However, secondary resistance mutations in SMO or genetic alterations of downstream HH target genes have been identified only in a subset of resistant MB and BCC tumors and failed to explain the emergence of resistance towards SMO inhibitors in other types of cancer. An alternative, intriguing hypothesis points to the existence of noncanonical, compensatory signal pathways that drive SMO-independent GLI1 activation in cancer, thus bypassing the inhibitory activity of SMO antagonists and hence contributing to the acquisition of resistance.

#### 6.3.1. PI3K/AKT/mTOR Pathway

The PI3K/AKT/mTOR signaling pathway is a key regulator of cellular processes, such as growth, proliferation, survival, and motility, and has been shown to inhibit the phosphorylation of GLI1 by GSK3β [177]. Studies suggested that this pathway increases GLI1 transcriptional activity and nuclear localization through phosphorylation of GLI1 at residue Ser84 by S6K1 [35], as well as by antagonizing PKA-dependent GLI inactivation [178], leading to an SMO-independent activation of HH pathway. The observation that heterozygous ablation of *PTEN* in mice carrying a SMO-A1 transgene promoted MB formation [179] strongly supports a role for the PI3K pathway in HH-driven MB (Figure 5, mechanism 4).

The first evidence of the involvement of the PI3K/AKT pathway in the emergence of resistance to SMO inhibitors came from a gene expression profiling of sonidegib-resistant vs. sonidegib-sensitive MB tumors. This screening identified a series of IGF1R-PI3K target genes upregulated only in the resistant ones [84]. The administration of the PI3K inhibitor NVP-BKMI20 in combination with sonidegib, from the time of initial treatment, significantly delayed the onset of resistance and subsequent tumor regrowth but failed to inhibit the growth of already established sonidegib-resistant tumors [84]. Treatment of vismodegib-resistant tumor models with the PI3K inhibitor GDC-0941 has been also shown to inhibit tumor growth [66].

As the compensatory upregulation of the PI3K pathway may contribute to the emergence of resistance to SMO inhibitors [66,84], the use of dual HH and PI3K/AKT/mTOR inhibitors could represent a promising approach to overcome resistance to SMO inhibitors, as previously reported [180].

#### 6.3.2. RAS-RAF-MEK-ERK Pathway 

The MAPK/ERK (mitogen-activated protein kinase/extracellular signal-regulated kinase) pathway (also known as RAS-RAF-MEK-ERK) has been identified as a way to evade SMO inhibition and drive tumor evolution in HH-dependent tumors. A previous study reported that this pathway regulates the nuclear localization and transcriptional activity of GLI1 in melanoma cells [28]. In another study, activation of the RAS/MAPK pathway in MB cell lines through the overexpression of HRAS-G12V and BRAF-V600E, but not that of PI3K/AKT, has been shown to induce resistance to SMO inhibitors sonidegib, LEQ-506, and vismodegib when transplanted in nude mice [174]. These studies suggest that activation of the RAS/MAPK pathway might circumvent HH pathway dependence rather than reactivate the HH pathway downstream of SMO (Figure 5, mechanism 5).

#### 6.3.3. Protein Kinases

Targeting post-transcriptional modifications of GLI1 has been recently proposed to overcome noncanonical HH pathway activation in tumors unresponsive or resistant to SMO inhibitors. Atwood et al. showed that the atypical protein kinase Cι/λ (aPKCι/λ) regulates both HH signaling and ciliogenesis in BCC. aPKCι/λ acts downstream of SMO to activate GLI1 through the phosphorylation of S243 and T304 residues in the zinc finger DNA binding domain of GLI1, maximizing its DNA binding ability [181]. The authors showed that SANT1-resistant BCC lines display increased aPKCι/λ levels and that the use of the aPKCι/λ inhibitor PSI is sufficient to prevent BCC progression in both vismodegib-sensitive and -resistant cells [181] (Figure 5, mechanism 6). 

The class I dual-specificity tyrosine phosphorylation-regulated kinase (DYRK) family members have been related to HH pathway activation. DYRK1A is able to increase transcriptional activity of GLI1 [182], and DYRK1B is involved in the autocrine-to-paracrine shift of HH signaling through upregulation of HH ligand expression [183]. Of note, DYRK1B has been recently identified as a possible therapeutic target to overcome resistance towards SMO inhibitors in GLI-dependent cancer cells. Indeed, DYRK1B drives noncanonical HH pathway activation by directly phosphorylating GLI1/2, thus enhancing the stability of their activator forms [184]. Importantly, both genetic and pharmacological inhibition of DYRK1B represses HH signaling in vismodegib-resistant cells (i.e., *SUFU*-deficient MB cells, pancreatic cancer cells, and Ewing sarcoma cells) [184] (Figure 5, mechanism 7). 

#### 6.3.4. Chromatin Modulators

Members of the histone deacetylase (HDAC) family were found to control the activity of the HH pathway by deacetylating GLI proteins. A previous study indicated that acetylation of GLI negatively impacts their DNA-binding capacity [185]. In support of this, the use of the selective HDAC1/2 inhibitor mocetinostat has been shown to inhibit the HH pathway in preclinical models of Shh MB through acetylation of GLI1 at the residue K518 [186]. Recently, Gruber et al. reported that treatment of human MB cells with class I HDAC inhibitor 4SC-202 strongly reduced the engagement of GLI1 to the promoter of its target PTCH1 and also increased the ratio of GLI3 repressor (GLI3R) to GLI3 full-length activator (GLI3A) in *PTCH1*-deficient mouse MB cells, also implying a role for acetylation in GLI processing. Importantly, the authors showed that administration of 4SC-202 was effective not only in vismodegib-sensitive MB cells but also in SUFU knocked-down resistant clones, suggesting that targeting the HH pathway at the level of GLI transcription factors with HDAC inhibitors could bypass the acquired resistance to SMO inhibitors [187]. Consistently, the dual HDAC/SMO inhibitor NL-103 has been reported to successfully overcome vismodegib resistance in SMO-M2 and SMO-D473H mutants by downregulated GLI2 expression [188], and coadministration of the HDAC inhibitor SAHA and vismodegib improved therapeutic outcomes for multiple aerodigestive cancer cell lines [189] (Figure 5, mechanism 8).

The bromo- and extra-terminal domain (BET) family of chromatin adaptors (BRD1–4 and BRDT in mammals) recognize and bind to ɛ-N-lysine acetylation motifs on open chromatin and facilitate gene transcription at super-enhancer sites across the genome by interacting with other proteins, including the positive transcription elongation factor b (P-TEFb) and RNA polymerase II (PolII) [190,191]. Within the BET proteins, BRD4 has been reported to regulate the transcription of GLI1 and GLI2 downstream of SMO and SUFU through direct occupancy of their promoters [192] (Figure 5, mechanism 9). Notably, treatment of BCC and MB with BET inhibitors JQ1 and I-BET151 was able to suppress the expression of HH pathway target genes even in the presence of resistance mechanisms to SMO inhibitors, such as mutations of *SMO* or *SUFU*, or *GLI2* amplifications [192,193], thus providing an effective strategy for treating resistant, HH-driven tumors.

#### 6.3.5. Other Mechanisms

Recently, Oro et al. investigated the mechanism of noncanonical activation of GLI1 in drug-resistant BCCs lacking activating mutations in SMO. Through a multidimensional genomics analysis using both mouse models for BCC resistance and human-derived tumors, they identified a key role for the transcription factor serum response factor (SRF) in the evolution of drug resistance. SRF has been shown to move into the nucleus in association with the transcriptional cofactor megakaryoblastic leukemia 1 (MLK1), where together they form a protein complex with GLI1 that enhances the transcription of HH pathway target genes and drug-resistant tumor growth [194].

Another case of noncanonical GLI1 activation is represented by loss of the component of the SWI/SNF chromatin remodeling complex SNF5 in malignant rhabDOId tumors, resulting in de-repression of transcriptional activity at GLI1 locus [39]. Similarly, the EWS-FLI oncogenic fusion gene has been reported to directly transactivate GLI1 in Ewing sarcoma [195].

The development of resistance towards SMO inhibitors in cancer has been also related to the existence of residual tumor cells that drive tumor regrowth with a mechanism that no longer relies on HH signaling for survival. For instance, Vanner et al. identified in MB a subpopulation of quiescent cells expressing high levels of the stem cell transcription factor SOX2, which is enriched in MB patient samples following treatment with vismodegib. These cells were unresponsive to vismodegib treatment and represented the propagating cells responsible for MB relapse from lineage tracking experiments, suggesting them as an attractive therapeutic target in combination with tumor debulking drugs to obtain a more durable MB remission [196]. SOX2 has been also reported to cooperate with the protein kinase Cι (PKCι) to drive tumorigenesis by establishing a cell-autonomous HH signaling axis in lung squamous cell carcinoma [197]. This suggests that the cotargeting of SOX2 and the HH pathway with SMO inhibitors could be a novel therapeutic approach to eradicate resistant cancer cells. In BCC, two recent studies demonstrated that treatment with vismodegib induces a cell identity switching that favors the selection of a subpopulation of quiescent residual tumor cells responsible for tumor relapse. These cells are characterized by a most permissive chromatin state that allows the activation of WNT signaling rather than de novo mutations [198], thus highlighting the importance of combination therapy with both SMO and WNT inhibitors to overcome tumor relapse in BCC [198,199].

## 7. Concluding Remarks

HH signaling is highly complex and plays important roles in promoting tumorigenesis, tumor progression, and drug resistance. Development of therapeutics for HH signaling has primarily focused on targeting SMO and, to a lesser extent, GLI. Except for ATO, which is not specific for GLI transcription factors, none of the GLI antagonists are good candidates for clinical studies because of their structure and chemical properties.

The increasing number of clinical trials using SMO inhibitors focus on the importance of targeting SMO in cancer. SMOi have already been found to be relatively effective in treating several types of cancer in preclinical studies and two of them received FDA approval for treatment of advanced or metastatic BCC. However, most of them showed limited efficacy in a number of cancer types or caused serious side effects in others. Therefore, further efforts must be made to limit adverse effects and to understand the mechanisms of resistance to small-molecule SMOi, finding novel strategies to overcome them. Several reports on BCC and MB patients indicate the occurrence of specific missense mutations in response to treatment with SMOi, in particular, SMO-D437H in vismodegib-treated patients. Further development of antagonists such as MRT-92, which bind multiple sites on SMO and show activity against the SMO-D473H mutant, could be crucial to overcome SMOi-induced resistance. In addition, MRT-92 has been shown to induce mitotic catastrophe in cancer cells, a desirable outcome for a novel anticancer drug. Furthermore, MRT-92 shows an excellent pharmacokinetic and metabolic profile and good tolerability in vivo with no sign of toxicity in preclinical models. Together, these data predict that MRT-92 will be a strong candidate for clinical trials and it could be further investigated, alone or in combination with other treatment strategies, for clinical efficacy on patients with HH-dependent cancers.

## Figures and Tables

**Table 1:** Oncogenic and resistance-associated SMO mutations.

Mutation	Role	Cancer Type	Reference
S278I	oncogenic	BCC, MB	[78,79]
L412F	oncogenic	BCC, Ameloblastoma, Meningioma	[78,80,81,82]
S533N	oncogenic	PNET	[83]
W535L (SMO-M2)	oncogenic	BCC, Ameloblastoma, Meningioma	[14,81,82]
R562Q (SMO-M1)	oncogenic	BCC	[14]
N219D (mN223D)	sonidegib-resistance	*Ptch*^+/−^; *p53*^−/−^ MB	[84]
L221R (mL225R)	sonidegib-resistance	*Ptch*^+/−^; *p53*^−/−^ MB	[84]
D384N (mD388N)	sonidegib-resistance	*Ptch*^+/−^; *p53*^−/−^ MB	[84]
S387N (mS391N)	sonidegib-resistance	*Ptch*^+/−^; *p53*^−/−^ MB	[84]
G453S (mG457S)	sonidegib-resistance	*Ptch*^+/−^; *p53*^−/−^ MB	[84]
H231R	vismodegib-resistance	BCC	[78]
T241M	vismodegib-resistance	BCC	[80]
W281C	vismodegib-resistance	BCC	[78,80]
V321M	vismodegib-resistance	BCC	[78,80]
I408V	vismodegib-resistance	BCC	[80]
L412F	vismodegib-resistance	BCC	[78,80]
A459V	vismodegib-resistance	BCC	[80]
F460L	vismodegib-resistance	BCC	[78]
C469Y	vismodegib-resistance	BCC	[80]
D473H*	vismodegib-resistance	MB	[65]
D473G*	vismodegib-resistance	BCC	[78,85]
D473Y	vismodegib-resistance	BCC	[86]
Q477E*	vismodegib-resistance	BCC	[78]
G497W	vismodegib primary res.	BCC	[86]
E518K/A	vismodegib-resistance		[66]
S533N	vismodegib-resistance	BCC	[80]
W535L*	vismodegib-resistance	BCC	[78,80,85]
Q635E	vismodegib-resistance	BCC	[78]

For location of these SMO mutations, see Figure 3. Sonidegib-resistance-associated SMO mutations were identified in an MB mouse model. *, indicates mutations, identified in vismodegib-resistant advanced BCC, that also reduce sonidegib efficacy [87]. BCC, basal cell carcinoma; MB, medulloblastoma; PNET, primitive neuroectodermal tumors; res., resistance.

**Table 2:** Inhibitors of the HH pathway.

Pathway Antagonists	Mechanism of Action	Status	Reference
***At SMO Level***			
Cyclopamine	Binds 7TM domain	Preclinical	[3]
KAAD-Cyclopamine	Binds 7TM domain	Preclinical	[3]
IPI-269609	Binds 7TM domain	Preclinical	[92]
GDC-0449 (Vismodegib)	Binds 7TM domain	68 Clinical trials	[98]
LDE-225 (Sonidegib)	Binds 7TM domain	37 Clinical trials	[99]
IPI-926 (Saridegib)	Binds 7TM domain	6 Clinical trials	[100]
BMS-833923/XL139	Binds 7TM domain	8 Clinical trials	[101]
PF-04449913 (Glasdegib)	Binds 7TM domain	11 Clinical trials	[102]
LY2940680 (Taladegib)	Binds 7TM domain	6 Clinical trials	[68]
Itraconazole	Binds SMO (BS distinct from Cyc)	48 Clinical trials	[103]
Posaconazole	Binds SMO (BS distinct from Cyc)	16 Clinical trials	[104]
TAK-441		1 Clinical trial	[105]
LEQ-506		1 Clinical trial	[67]
Vitamin D3	Binds 7TM domain	3 Clinical trials	[73]
Cur-61414		Preclinical	[106]
PF-5274857		Preclinical	[107]
Compound 5		Preclinical	[66]
SANT1-4	Bind 7TM domain	Preclinical	[60]
ALLO 1-2	Bind extracellular CRD	Preclinical	[108]
DMB5	Binds 7TM domain	Preclinical	[109]
MRT-83	Binds 7TM domain	Preclinical	[110]
MRT-92	Binds 7TM domain	Preclinical	[111,112]
SA1-10	Inhibit SMO ciliary localization	Preclinical	[113]
Budesonide	Inhibits SMO ciliary translocation	Preclinical	[114]
SMANT	Inhibits SMO ciliary translocation	Preclinical	[115]
DY131	Inhibits SMO ciliary translocation	Preclinical	[115]
Smoothib		Preclinical	[116]
HH78		Preclinical	[117]
A8		Preclinical	[118]
SEN450		Preclinical	[119]
BRD-6851		Preclinical	[120]
Benzamide derivatives		Preclinical	[121,122]
Tetrahydropyridopyrimidine derivatives		Preclinical	[123]
Tetrahydrothiazolopyridine derivatives		Preclinical	[124]
Quinazolinone derivatives		Preclinical	[125]
Phenyl imidazole derivatives		Preclinical	[126]
Piperazine-1-carboxamides		Preclinical	[127]
Piperazinyl urea derivatives			[128]
N-arylpropanamide		Preclinical	[129]
Benzimidazole derivatives		Preclinical	[130]
***Downstream of SMO***			
GANT58-61	Inhibit GLI-mediated luciferase	Preclinical	[131]
Arcyriaflavin C	Inhibits GLI-mediated luciferase	Preclinical	[132]
Physalin F	Inhibits GLI-mediated luciferase	Preclinical	[132]
HPI1-4	Modulate GLI activation	Preclinical	[133]
ATO	Inhibits GLI transcription factors	41 Clinical trials	[134,135]
Pyrvinium	Enhances GLI degradation	Preclinical	[136]
Glabrescione B	Interferes with DNA binding	Preclinical	[137]

ATO, arsenic trioxide; SMO, Smoothened; 7TM, heptahelic transmembrane domain; CRD, cysteine rich domain; BCC, basal cell carcinoma; BS, binding site; Cyc, cyclopamine. Status: preclinical or in clinical trials (http://clinicaltrials.gov) assessed on 25^th^ October 2018.

## Abbreviations

HH	Hedgehog-GLI
SMO	Smoothened
BCC	Basal cell carcinoma
MB	Medulloblastoma
PNET	Primitive neuroectodermal tumors
AML	Acute myeloid leukemia
SHH	Sonic hedgehog
IHH	Indian hedgehog
DHH	Desert hedgehog
PTCH1	Patched 1
GPCR	G-protein-coupled receptor
SUFU	Suppressor of Fused
KIF7	Kinesin family member 7
PC	Primary cilium
PKA	Protein kinase A
CK1	Casein kinase 1
GSK3β	Glycogen synthase kinase 3β
HHIP1	Hedgehog-interacting protein
TNF-α	Tumor necrosis factor-α
mTOR	Mammalian target of rapamycin
S6K1	S6 kinase 1
ECD	Extracellular domain
CRD	Cysteine-rich domain
TMD	Transmembrane domain
ICL	Intracellular loop
ECL	Extracellular loop
Gprk2	G protein-coupled receptor kinase 2
PI4P	Phosphatidylinositol 4-phosphate
Cul4	Cullin4
DDB1	DNA damage binding protein 1
DBP	Drug-binding pocket
FDA	Food and Drug Administration
Pgp	P-glycoprotein
ATO	Arsenic trioxide
IGFR	Insulin growth factor receptor
PTEN	Phosphatase and tensin homolog
PI3K	Phosphatidylinositol-4,5-bisphosphate 3-kinase
EGFR	Epidermal growth factor receptor
PDGFR	Platelet-derived growth factor receptor
MAPK	Mitogen-Activated Protein kinase
ERK	Extracellular Signal-Regulated Kinase
aPKCι/λ	Atypical protein kinase Cι/λ
DYRK	Dual specificity tyrosine-phosphorylation-regulated kinase
HDAC	Histone deacetylase
BET	Bromo- and extra-terminal domain
SRF	Serum response factor
MLK1	Megakaryoblastic leukemia 1
